# Determinants of paying national health insurance premium with mobile phone in Ghana: a cross-sectional prospective study

**DOI:** 10.1186/s12939-019-0946-x

**Published:** 2019-03-25

**Authors:** Joseph Marfo Boaheng, Eugenia Amporfu, Daniel Ansong, Anthony Kofi Osei-Fosu

**Affiliations:** 1 Faculty of Social Sciences, Department of Economics, Kwame Nkrumah University of Science and Technology, Kumasi, Ghana; 2 Department of Child Health, Kwame Nkrumah University of Science and Technology School of Medicine and Dentistry, Kumasi, Ghana

**Keywords:** Enrollment, Retention, National Health Insurance Authority, National Health Insurance Scheme, Mobile phone payment system

## Abstract

**Introduction:**

In an effort to increase Ghana’s National Health Insurance Scheme (NHIS) enrollment and retention rates, the NHIS introduced membership renewal and premium payment by mobile phone. The success of such an innovation dependents on many factors including personal and community characteristics of members.

**Objective:**

The objective of the study is to investigate the determinants of renewing membership and paying the NHIS premium through a mobile phone.

**Methodology:**

The prospective cross-sectional survey was used to solicit the required information from about 1192 respondents living in Kumasi Metropolis, Atwima Nwabiaya and Sekyere Central Districts of Ghana. Logistic regression model was employed to estimate the determinants of paying the NHIS premium with the mobile phone.

**Results:**

The study found that factors including *residing in an urban area (Kumasi metropolis), senior high education, tertiary education and informal employees* are the determinants of paying the NHIS premium with the mobile phone.

**Conclusion:**

It is recommended that the NHIS consider making the mobile payment as simple as possible for the less educated and the rural members to access it.

## Introduction

In December 2018, the Ghanaian National Health Insurance Scheme (NHIS) introduced an innovative method of membership renewal and premium payment through the mobile phone. The new system uses the mobile money banking system to allow members to renew their membership on any of the mobile money platforms [1]. This initiative is part of the effort of the NHIS to totally digitize enrollment and renewal [1]. With this method, members can receive alert when their membership is due for renewal. It is thus expected to increase enrollment on the NHIS.

The NHIS was introduced in Ghana in 2003 to replace the existing out-of-pocket fee-paying system locally known as Cash and Carry. Under the Cash and Carry system, patients paid for healthcare provided at the point of purchase. While the Cash and Carry might have provided the much-needed revenue to run health facilities in the health sector, it imposed a financial burden on the poor with the accompanied negative impact on health due to underutilization of care [2]. Under the NHIS, members do not make copayment and so the Scheme provides financial risk protection to its members. However, enrollment on the NHIS has been slower than expected, with the current coverage being about 40% of the population, implying that the NHIS does not even cover half of the population.

Currently, registration and renewal of membership involve travelling to the NHIS designated areas for the service and this may impose a high travel cost on members in terms of money, time, and the inconvenience. There have been many studies on the factors affecting enrolment on the NHIS. Some of the factors identified as impeding growth in enrollment include poverty, quality of care in NHIS accredited facilities, culture, inadequate distribution of social infrastructure, religion, among others [3, 4]. None of these studies however included transportation and time cost for renewal or registration of the insurance as factors. According to the NHIS, the introduction of membership renewal by mobile phone was to relieve the NHIS members of the stress of spending long period at NHIS offices for registration and renewal [5]. The use of mobile phone for membership renewal and premium payment is expected to ease membership renewal and improve enrollment as well as retention of membership. The electronic renewal system was piloted in a district each in the Northern and Eastern regions [1]. The policy raises a lot of questions including factors that ensure its success. The purpose of this study is to find the determinants of NHIS members’ patronage of the electronic renewal of their membership via mobile phones in the Ashanti region of Ghana.

### The NHIS in Ghana

The membership of the NHIS is categorized into eight: those in the informal sector, those under 18 years old, the 70-year old and older, Social National Insurance Trust (SNNIT) contributors, SNNIT pensioners, pregnant women, indigents, and members under the Livelihood Empowerment Against Poverty (LEAP). With the exception of those in the informal sector, none of the members pay premium. However, all need to register for membership and renew their membership every year. Under the initiative, all categories can renew their membership by mobile phone, while those in the informal sector also renew their membership and make payment of premium by mobile money [1].

Ghana’s vision to alleviate the burden of healthcare cost of its citizens was realized in 2003 when the NHIS was introduced. The core objective of the Scheme is to provide equity in the health sector as well as ensure affordable healthcare for the poor [6], an objective which is consistent with the universal health coverage (UHC). The NHIS is then an instrument for the achievement of UHC in Ghana [6]. The Sustainable Development Goal 3.8 is the achievement of UHC with financial risk protection and access to quality essential health services as well as medicines and vaccines. It is important for the NHIS to expand both enrollment and retention rates of membership in order to provide the needed financial risk protection to all because the annual growth rate of enrollment on NHIS is only about 1% [7, 8].

Some studies done on the NHIS reveal that the insurance premium may not be affordable to some members in the informal sector under the current payment system due to irregularity in their income streams [9, 10]. The pre-mobile phone payment system (MPPS) is characterized by non-flexibility in insurance payment, transportation time and cost, long waiting time in queues at the NHIS offices. These problems arising from such payment method could explain why enrollment and retention onto the scheme are low for people in the informal sector. For the NHIS members in the informal category, membership is a function of premium, therefore, affordability of the premium can predict the enrollment or retention rates on the insurance scheme. One of the potential advantages of paying premium by mobile phone is that it is possible to introduce flexibility in payment to allow those who would normally not afford to pay their premium up front to make gradual payments for membership on the NHIS. It is therefore important to identify the factors that determine the success of the mobile phone renewal and payment system to ensure its success. Previous studies on factors affecting enrollment on the NHIS identified age, gender, health status, income status, availability of health facility, marital status, culture, and religion among others [3, 4]. It is important to find out the extent to which these factors could also affect the patronage of the mobile phone renewal membership and payment of premium.

### Mobile phone utilization

There has been a rapid increase in mobile phone penetration globally particularly in the Asia Pacific region, with 90% of the global and 80% of the rural population having access to a mobile network in 2010 [11]. In that same year, the number of subscriptions reached 5.3 billion following earlier study in 2006 that reported an estimated subscription of 2 billion [12]. This represents 76.2% global penetration rate. In Africa, the demand for mobile phones is huge and rapidly increases having recorded 50milliion subscribers in the past decade [13]. This represents 7% of the continent’s population. With an annual growth rate of 35% according to Scott et al., [13], it is expected that the current figure will overwhelmingly increase after 10 years.

The consideration of mobile phone for NHIS membership renewal in Ghana is as a result of extensive use of mobile phones in Ghana. Available statistics by Ghana’s National Communication Authority (NCA) indicate that the number of registered active mobile phone chips in Ghana as of December 2017 stands at 36.75 million. With a population of about 28.83 million in 2017 this is an indication that on the average, each Ghanaian has 1.27 mobile phones. Mobile phones are extensively utilized by the population in both the formal and informal sectors, and the utilization is expected to increase. The number of registered active mobile phone is projected to increase to 40 million while population is projected at 31 million in 2020 [14].

The use of mobile phones in the health sector is not new but not much studies have been done to identify the factors affecting their success. Earlier studies have shown how mobile phones have played remarkable roles in healthcare delivery. For instance, text messaging interventions have been adopted to improve compliance to medication and to clinical appointments [15–18]. The NHS Direct which is a nurse-led telephone service provides basic medical advice to callers 24 h a day, 7 days a week in the UK [19]. Analysis of the calls showed that the service was patronized by male callers between 16 and 44 years [19], implying that age and gender play an important role in the success of the program. A study that reviewed the literature on the use of mobile phone messaging interventions to support and improve preventive healthcare, health status and health behavior outcomes showed that only some selected interventions were successful [20]. Information on users’ satisfaction which is an important determinant of the success of the intervention is unknown, A similar study also reports limited evidence that mobile phone messaging interventions are likely to provide benefit in supporting the self-management of long-term illnesses [11]. The intervention involves the use of Short Message Service (SMS) and Multimedia Message Service (MMS) to improve patients’ self-efficacy skills through medication reminders and therapy adjustments or supportive messages [11]. In Malawi, HIV and AIDS patients receive text messages daily to remind them about their medication schedule [21]. Again, there is little evidence of the acceptability of the interventions. Furthermore, mobile phones have also been employed through SMS to support college students to quit smoking, but studies show that further research is needed to determine its success [22].

None of the above interventions involved the use of mobile phone for healthcare financing. Ghana is however not the first to use mobile phone for health insurance membership renewal. Kenya has made a bold step by launching a mobile phone platform called “M-Pesa” (Mobile Money Payment System) for paying the health insurance premium on a monthly basis [23]. The method of payment has become increasing popular among members of Kenya’s National Health Insurance [23]. However, no study has been done on its patronage. In the case of Ghana, it is only now that the mobile money system is considered an avenue to pay for health care bills in some health facilities especially in private health care providers.

As already mentioned, there has been no study on the factors that determine the patronage in the use of mobile phone for renewal of membership and payment of premium of insurance. Just as characteristics of members were identified as being important in affecting the enrolment on the NHIS, the current study expects members’ characteristics to be important determinants of the patronage of the innovation. In accordance with the previous studies, factors to be examined include age, gender, education level, employment status, marital status, area of residence, and distance between residence and NHIS office. Previous studies [3] have shown that females, older adults, the employed, married, and urban dwellers are likely to enroll on the NHIS. However, de Jongh et al., [11] has shown that young males are likely to patronize services that require the use of mobile phone. The current study then examines the role of these characteristics in affecting the patronage of the mobile phone payment method and hence effect on enrolment on the NHIS.

## Methodology

### Model specification

The model is specified below;

$$ {\displaystyle \begin{array}{l}{y}_i={\beta}_0+{\beta}_1 AGE+{\beta}_2 LOC+{\beta}_3 FEMALE+{\beta}_4 MARITAL+{\beta}_5 EDU\\ {}\kern1.75em {\beta}_6 EMPL+{\beta}_7 DEP+\varepsilon \end{array}} $$

Where *y*_*i*_ is a binary variable which equals 1 if respondent is willing to pay premium through mobile phone and zero otherwise. AGE is a vector of three age dummy variables with one variable equaling 1 if the respondent is aged between 19 and 44 years and zero otherwise. The other age dummy variable equaled 1 if the respondent is between 44 and 69 years and zero otherwise. The final age dummy variable equaled 1 if the respondent is above 70 years and zero otherwise. LOC is a vector of two variables with one dummy variable which equaled 1 if the respondent lived in Urban (Kumasi) and zero otherwise. The other dummy variable equaled 1 if the respondent lived in the rural area and zero otherwise. EDU is a vector of four dummy variables for no education, primary education, secondary education and tertiary education. The no education dummy variable equaled 1 if the respondent has no educational background and zero otherwise. By similar reasoning primary, secondary and tertiary are for those with primary, secondary and tertiary education respectively. FEMALE equaled 1 for female respondents and zero for males. MARITAL is a vector of three dummy variables. One dummy variable equaled 1 for married respondent and zero otherwise, the second dummy variable equaled 1 for respondents who have formerly married and zero otherwise, while the other equaled 1 for respondents who were single and zero otherwise. EMPL is a vector of three dummy variables with one dummy variable for respondents who work in the formal sector, another dummy variable for respondents who are formal sector employees and the other for those who are unemployed. Finally, DEP is also a continuous variable that measured the number of dependents of respondent. Logistic Regression method of estimation was used to estimate the factors that influenced the decision of individuals to renew their membership of NHIS and pay premium with the mobile phone payment system.

### Methods

The paper adopted a prospective cross-sectional survey. The survey collected data from 1195 respondents to estimate factors that would affect paying health insurance premium via Mobile Phone Payment System. The respondents who participated in the study were insured in NHIS aged 18 years or older. This is due to the fact that in order to qualify to pay the NHIS premium in Ghana, the person has to be at age 18 years and older. With the aid of a standardized electronic questionnaire, the paper employed 10 enumerators to elicit the required data needed to address the objective of this study after each participant has consented to participate in the study. The survey collected information on the characteristics of the respondents including age, gender, income, area of residence, level of education, number of dependents, employment status, distance between residence to NHIS office, marital status, and affordability of premium.

In determining a sample size for this paper, a proportion *P*, of individuals who were willing to renew their membership and pay the NHIS premium with mobile phones from a pretested study was estimated at 75%. The paper also assumed a two-tail test, implying that a sampling error, *d* = 0.025 and precision, *z* = 1.96 were needed for a sample size determination. Taking into account the total population of the three selected districts, *N* = 2, 255, 321 and using the formula, $n={z}^{2\ast}\frac{p\left(1-p\right)}{d^2+\left({z}^{2\ast }p\left(1-p\right)/N\right)}$ [24], a minimum sample size, *n* of 1152 was required for the study. Based on the population size of each of the chosen districts, the sample sizes 974, 120 and 57 were allocated to Kumasi Metropolis, Atwima Nwabiagya and Sekyere Central districts respectively. The study further employed cluster sampling technique to select these participants from 20 clusters in the 3 districts chosen. The clusters in this paper represented the communities in the selected districts. Considering the population sizes of the individual districts, 15 clusters were selected from Kumasi Metropolis, and 5 from Atwima Nwabiagya and Sekyere Central. The Probability Proportional to Scale methodology was employed to identify the clusters from each district.

The current study was carried out in urban and rural district in Ashanti Region of Ghana (the largest region in terms of population). The choice of these categories of districts was to ensure adequate representation of participants with different characteristics in the study settings. The region’s population as of the 2010 census stood at 4,780,280, representing 19.4% of the country’s total population [25]. The district representing the urban stratum was Kumasi Metropolis, followed by Atwima Nwabiagya. Sekyere Central District was classified as a rural district in this study.

The Kumasi Metropolitan Assembly, represents 36.2% of the total population of Ashanti Region [25]. Out of the population 12 years and older, 72.4% own mobile phones [25]. The Atwima Nwabiagya District, on the other hand, has a population of 149,025, with a mobile phone usage for the population 12 years and older, of 58.6% have mobile phones [25]. The Sekyere Central District with a population of 71,232 represents 1.5% of the region’s total population. Of the population, 12 years and above, 28.1% have mobile phones [25].

## Results and discussion

### Descriptive results

The socio-demographic characteristics of the respondents were age, sex, marital status, number of dependent education, employment, location and premium affordability were analyzed. The chi-square test of association was also conducted to test the hypothesis that the relationship between the socio-economic variables and renewal of NHIS membership plus premium with the mobile phone is zero. The findings of the descriptive results are presented in Table 1.

**Table 1:** Descriptive Statistics

Variable	Category	Frequency	Percentage
Mobile Phone usage	Yes	1159	96.99
	No	36	3.01
Age in categories	Young Adult (18–44)	876	73.31
	Middle Adult (45–69)	311	26.03
	Older Adult (70+)	8	0.67
Location	Kumasi metropolis (KMA)	1038	86.86
	Atwima Nwabiagya (AN)	117	9.79
	Sekyere central (SC)	40	3.35
Gender	Female	619	51.80
	Male	576	48.20
Marital Status	Married	635	53.14
	Formerly married	149	12.47
	Single	411	34.39
Education	No education	40	3.35
	Basic education	374	31.30
	SHS	500	41.84
	Tertiary education	281	23.51
Employment	Unemployed	155	12.97
	Formally employed	173	14.48
	Informally employed	867	72.55
Age	Mean (SD)	36.39 (11.51)	
Dependents	Mean (SD)	1.94 (1.91)	

The descriptive results depict a greater proportion of the sampled population (96.99%) use mobile phones. All the respondents fell in the 18 to 75 age range, with 36.39 years (SD = 11.51) as the mean age. Within the age category, the younger age forms the majority (73.31%) while the older adult group was the minority (0.67%). A higher proportion of respondents representing 86.86% reside in urban area of Kumasi Metropolis while the minority (3.35%) lives in rural sekyere central district. Out of the 1195 individuals sampled, 51.80% were females. The results also indicate that about 53.14% forming the highest percentage had married. As regards marital status, those who had formerly married were the category with the least representation (12.47%). On education, a greater proportion of the respondents (41.84%) had completed SHS/MSLC followed by tertiary level representing about 23.51%. Those without any form of educational background were the least (3.35%) in the sample. Furthermore, the survey showed average dependents of 1.94, which may translate into 2 dependents per respondent since a person cannot be halved. The respondents’ employment was another important variable considered. Most of the respondents were employed in the informal sector (72.55%) while the unemployed forming the minority represented 12.97%.

### Model diagnosis

#### Goodness-of-test

From the result of likelihood ratio test for the overall model evaluation (see Table 3), the null hypothesis which states that the model without independent variables provides a better fit to the data than the model with independent variable was rejected. This implies that at least one of the independent variables contributed to the prediction of an individual’s decision to pay NHIS premium with the mobile phone [26]. Also, in examining whether the observed proportions of events are similar to the predicted probabilities of occurrence in subgroups of the model population, the Hosmer-Lemeshow test prescribed by [27] was conducted (see Table 3). The results suggest that the null hypothesis which states that the model fits the data well was not rejected. This implies that our model passed the test.

#### Predictive accuracy and discrimination

The classification table by [28] employed in this analysis evaluated the predictive accuracy of the logistic regression model. Using a cutoff value of 0.5, all predicted values above 0.5 were classified as predicting the likelihood of paying health insurance premium with mobile phone from a set of covariates and all below 0.5 as otherwise. From Table 2, the overall percentage correct value (94.18%) showed an increased predictive accuracy of an individual’s decision to pay NHIS premium through mobile phone given some covariates. The sensitivity and specificity values indicate a better fit of the model [26]. The sensitivity results indicated that the probability of the model to correctly identify those who are likely to pay NHIS premium with the mobile phone was 99.80%. Out of this, the predictive positive value of 94.33%, denotes the actual number of people who shall pay with the mobile phone. On the other hand, the specificity results show that the probability of the model to correctly identify those who are not likely to pay NHIS premium with the mobile phone was 9.23%. Out of this, the negative predictive value of 75.00% represents the actual number of people who shall not pay with the mobile phone.

**Table 2:** Discrimination Test with a Classification Table

Observed	Predicted		Total	% Correct
	Yes	No		
Yes	981	59	1040	
No	2	6	9	
Overall % Correct	983	65	1048	94.18

Sensitivity = 99.80%, Specificity = 9.23%, Positive predictive value =94.33%, Negative predictive value = 75.00%

**Fig. 1:**
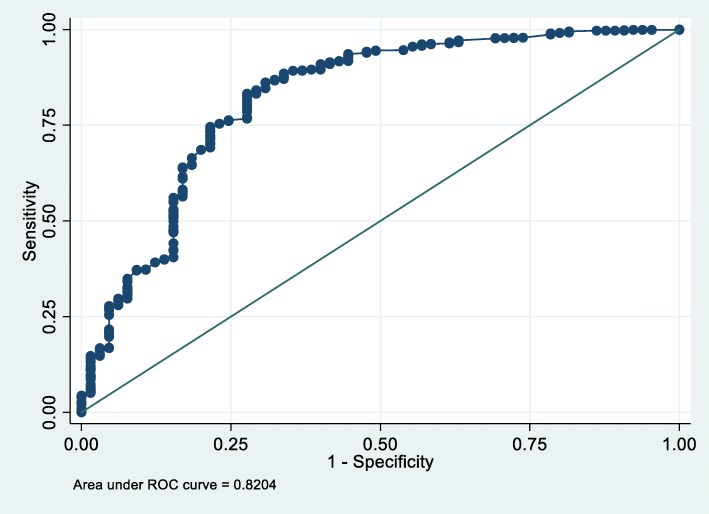
ROC of PAYING Insurance Premium with Mobile Phone

To probe further, the full range of threshold values ranging from 0 to 1 were examined with Receiver Operating Characteristics (ROC) curve unlike the classification table of just selecting a single threshold point (see Figure 1 in the Appendix). The ROC better discriminates those who shall pay NHIS premium with mobile phone based on some determinants against those who will do otherwise. The result showed that the larger Area Under the Curve (AUC) i.e. 82% and above 45^0^ line for the variables representing *age, KMA (urban), female, marital status, education and employment * depicted better predictability of the model. This finding agrees with the assertion that larger AUC and points above and further away from 45^0^ line indicate better predictability of a model [29].

### Regression results

The findings from the logistic regression model presented in Table 3 indicate that the variables representing *urban (KMA), senior and tertiary education, informal and formal employees* were significant predictors for an individual’s decision to pay NHIS premium with the mobile phone in Ghana.

**Table 3:** Logistic Results of Determinants of Paying NHIS Premium by MPPS

Paying by Mobile Phone	OR	S.E.	Z	P>z	95% C.I.	
Young Age	2.1436	2.2888	0.7100	0.4750	0.2644	17.3780
Middle Age	1.4251	1.5304	0.3300	0.7410	0.1737	11.6935
KMA	15.8932***	4.9820	8.8200	0.0000	8.5978	29.3789
Female	1.7828	0.5345	1.9300	0.0540	0.9906	3.2086
Married	1.3099	0.4280	0.8300	0.4090	0.6904	2.4851
Income	3.0665	2.1316	1.6100	0.1070	0.7852	11.9766
Formerly married	2.4325	1.6846	1.2800	0.1990	0.6260	9.4525
Basic Education	4.1707*	3.0025	1.9800	0.0470	1.0173	17.0995
Senior High School	4.8877*	3.7964	2.0400	0.0410	1.0665	22.4000
Tertiary Education	2.4419*	0.9311	2.3400	0.0190	1.1565	5.1559
Informally employee	4.7950*	3.1220	2.4100	0.0160	1.3384	17.1789
Formally employee	1.0903	0.0885	1.0700	0.2860	0.9300	1.2783
Constant	2.1436	2.2888	0.7100	0.4750	0.2644	17.3780
No. of Observation = 1040						
R^2^ = 0.2316						
Test	Categories		*X* ^*2*^		P-Value	
Overall model evaluation	Likelihood ratio test		-187.23135		0.0000	
Goodness-of-fit test	Hosmer & Lemeshow		11.95		0.1539	

*p < 0.05; ***p < 0.001

The odds ratio of the *KMA variable* indicates that residents of Kumasi Metropolis are more than 15 times as likely to pay health insurance premium by mobile phone than those in rural districts, holding all other variables constant (OR = 15.89, *p <* 0.0001, C.I. = 8.5978–29.3789). The odds of the *basic* (OR = 4.17, *p* = 0.0470, C.I. = 1.0173–17.0995)*, secondary* (OR = 4.89, *p* = 0.0410, C.I. = 1.0665–22.4000) and *tertiary education* (OR = 2.44, *p* = 0.0190, C.I. = 1.1565–5.1559) variables depict that the respondents with secondary, and tertiary education levels are more than 4.17, 4.89 and 2.44 times as likely to pay NHIS premium through the MPPS than those without any form of education all other variables held constant. Finally, the significant effect and odd ratios of informal (OR = 4.80, *p* = 0.0160, C.I. = 1.3384–17.1789) employees show that those individuals are more than 4.8 times as likely to pay NHIS premium with their mobile phones than the unemployed assuming that all other variables remain the same.

### Discussion

From the logistic regression estimates in Table 3, the variable representing *urban area* i.e. *Kumasi Metropolitan Area (KMA), education –basic, secondary & tertiary and informal employee* were significant predictors for an individual’s decision to pay NHIS premium with the mobile phone. The result that residents of *urban* area are more likely to renew their membership and pay the NHIS premium with their mobile phone than those in rural area was expected. This is because urban areas have better infrastructure for the use of mobile phone than rural areas. People in the urban areas are more likely to use mobile phones conveniently and more frequently than individuals in rural communities. Access to mobile phones shops to purchase phone and recharge credits is easier and convenient in the urban centres than in the rural areas. Besides, NHIS renewal rates are higher in urban areas than rural areas [30], and urban population exceeds that of given rural area, urban membership renewal centers maybe more overcrowded than rural centres, hence the greater willingness of urban dwellers to patronize the use of mobile phone for renewal.

The result also showed that respondents with educational background were more likely to patronize the use of mobile phone for NHIS renewal than the uneducated. Such results were expected because the educated are more likely to understand the use of mobile phone than the uneducated. Besides, the educated are more likely to enroll and renew their membership on the NHIS than the uneducated [31] and so are likely to value the improved efficiency in membership renewal that results from the use of mobile phone. This, therefore, depicts that health insurance enrollment and retention through the MPPS is more likely to increase in a population with higher educational background.

The empirical results also showed that both *informal employees* are more probable to renew and pay the NHIS premium with mobile phones than the unemployed. This finding was expected because the employed have higher opportunity cost from waiting in long queues to renew their membership than the unemployed. The introduction of renewal through mobile phone significantly reduces such cost and hence makes it convenient for them to enroll and renew their membership. This is important because those in the informal sector pay premium and so the convenience of renewal by mobile phone could retain and even increase enrolment of employees from the informal sector who bring in new revenue. Even though individuals who are employed may be likely to be insured than the unemployed [32, 33], however in Ghana, the unskilled and agricultural workers who are often found in the informal sector are less likely to enroll in the NHIS [34]. Thus, the introduction of NHIS membership renewal by mobile phone could increase enrolment.

Finally, the results showed that age, female and marital status did not affect the decision to renew one’s NHIS membership by mobile phone. Even though the literature has shown that the youth spend more time on their mobile phone than the older population [35], the current study has shown that with regard to the renewal of NHIS membership by mobile phone, the willingness for patronage does not vary with age. The literature has shown that women spend more time on their mobile phone than males [35] and so may prefer using their mobile phones for transactions than males. Besides, females are more likely to enroll and renew their membership on the NHIS than males [36, 34]. However, the current study shows that the willingness to renew membership by mobile phone does not vary with gender.

## Conclusion and recommendation

The study has shown that there is a high patronage of the option for membership renewal by mobile phone, but the patronage varies depending on the characteristics of the individual. The traditional method of membership registration and renewal which involves queueing at the office of the National Health Insurance Scheme’s (NHIS) has been deemed inefficient and so the NHIS has introduced the option to renew membership by mobile phone. The current study examined the determinants of the patronage of the new option. The results showed that the main determinants are location of residence, gender, education, and employment status of the individual. The likeliness of patronage, however, did not vary with age, income, and marital status. The sample size and the restricted study area are the main limitations of the study. Future study could include the other regions of Ghana to make the data more representative of the nation. Nevertheless, the current study initiates an important area of study that is necessary to ensure the success of the new intervention.

Based on the findings, the study makes three recommendations to ensure the success of the intervention. First, the government should ensure that phones and their accessories are easily accessible in rural areas. This can improve patronage in the rural areas. Secondly, campaigns for patronage of the intervention need not target age groups or wealth status but should target location of residence, individuals with basic, secondary or tertiary education, and the employed especially the informal sector employees as they are likely to patronage the intervention. Thirdly, effort is needed to attract the uneducated, the unemployed, the formal employees and both females and males to adopt the intervention. Making the process as user friendly and as simple as possible could motivate many who otherwise would not patriciate to do so.

## Abbreviations

KMA: Kumasi Metropolitan Assembly
MMS: Multimedia Message Service
MPPS: Mobile Phone Payment System
NHIA: National Health Insurance Authority
NHIS: National Health Insurance Scheme
SMS: Short Message Service
